# Right atrial strain in Anderson–Fabry disease

**DOI:** 10.3389/fcvm.2025.1496534

**Published:** 2025-02-18

**Authors:** Rosa Lillo, Alessio Cianci, Maria Chiara Meucci, Giulia Iannaccone, Claudio Di Brango, Filippo Tusa, Mario Marsilia, Gaetano Antonio Lanza, Antonella Lombardo, Francesco Burzotta, Francesca Graziani

**Affiliations:** ^1^ Dipartimento di Scienze Cardiovascolari-CUORE, Fondazione Policlinico Universitario A. Gemelli IRCCS, Rome, Italy; ^2^ Facoltà di Medicina e Chirurgia, Dipartimento di Scienze Cardiovascolari-CUORE, Università Cattolica del Sacro Cuore, Rome, Italy

**Keywords:** Anderson–Fabry disease, right atrium, speckle tracking echocardiography, strain, cardiomyopathy

## Abstract

**Background:**

To date, only limited data are available on right atrium (RA) morphofunctional remodeling in Fabry disease (FD).

**Purpose:**

We aimed to investigate RA structural and functional remodeling in patients with FD vs. healthy controls using 2D speckle tracking echocardiography (STE) and to explore whether any differences exist in FD patients with and without left ventricular hypertrophy (LVH).

**Methods:**

We prospectively enrolled patients with FD and controls matched for age, sex, and cardiovascular risk factors. Patients with FD were divided in two groups according to the presence/absence of LVH (LVH+: left ventricular wall thickness >12 mm). All patients underwent standard echocardiography and STE analysis investigating the mechanics of all cardiac chambers, including RA reservoir, contractile and conduit strain.

**Results:**

A total of 64 patients with FD (50% males; mean age 50 ± 17 years; 51.5% LVH+) and 64 control patients were included in the study. Focusing on right chambers, RA and right ventricular (RV) dimensions were similar between FD and controls. No differences were found for tricuspid annular plane systolic excursion (*p* = 0.073) and RV fractional area change (*p* = 0.461), while RV systolic Tissue Doppler velocity was reduced in patients with FD (*p* = 0.041). STE analysis revealed impaired strain values for all cardiac chambers in FD vs controls, specifically: left ventricular global longitudinal strain (LV-GLS, *p* < 0.001), left atrial (LA) reservoir strain (*p* = 0.001), conduit strain (*p* = 0.012), and contractile strain (*p* < 0.001), RV-GLS and RV free wall strain (*p* < 0.001). Similarly, all RA strain phases were significantly reduced in patients with FD compared with control patients (RA reservoir 27.4 ± 11.1 vs. 41.9 ± 8.3%, *p* < 0.001; RA contractile 9.9 ± 5.1 vs. 18.0 ± 4.9%, *p* < 0.001; RA conduit 19.1 ± 8.1 vs. 24.1 ± 8.1%, *p* = 0.001). When comparing FD patients without LVH to controls, it was found that RA reservoir and contractile strains were significantly reduced in the former (*p* < 0.001). In multivariable linear regression analyses, LA reservoir strain (*p* = 0.010) and LV-GLS (*p* = 0.044) emerged as independent correlates of RA mechanics after adjustments were made for RA dimensions, RV systolic function parameters and hypertrophy, and LV maximal wall thickness.

**Conclusions:**

In FD impaired RA strain is a common finding. RA reservoir and contractile strains are reduced in FD patients even before LVH ensues, as compared to controls. LA reservoir strain and LV-GLS show an independent correlation with RA reservoir strain.

## Introduction

Anderson–Fabry disease (FD) is a rare X-linked lysosomal storage disorder caused by pathogenic variants of the α-galactosidase A gene, resulting in complete or partial deficiency of α-galactosidase A (α-Gal A) enzyme activity and consequent globotriaosylceramide (Gb3) accumulation (1). Overt cardiac involvement is usually defined by the presence of left ventricular hypertrophy (LVH) (2), but histological studies have proved that all cardiac chambers are affected by Gb3 storage (3, 4).

Two-dimensional speckle tracking echocardiography (2D-STE) is a powerful tool for the assessment of cardiac chamber mechanics, and the evaluation of LV global longitudinal strain (LV-GLS) is widely used in clinical practice. Also right ventricle (RV) and left atrium (LA) strains are emerging as novel markers of dysfunction in cardiomyopathies (5–8), and their impairment is linked to prognosis (9, 10). In the context of Fabry cardiomyopathy, LV (11) and RV strains (5) have already been investigated, as also LA strain (7, 12). Specifically, LA deformation can be impaired even before the occurrence of LVH and overt diastolic dysfunction (13). To date, only limited data are available on right atrium (RA) remodeling in FD. Therefore, our aim in this study is to assess RA structural and functional remodeling in patients with FD compared with sex and age-matched healthy controls (HCs) and to explore whether any differences exist in FD patients with and without LVH.

## Materials and methods

### Study population

This is a prospective study performed at the Cardiac Rare Disease Outpatient Clinic, Fondazione Policlinico Universitario A. Gemelli IRCCS, Rome, Italy, between July 2020 and March 2024. All patients with a diagnosis of FD were screened (*n* = 78). We excluded patients whose speckle tracking analysis was not feasible due to poor image quality (*n* = 14). FD was diagnosed by measuring plasma and leucocyte alfa-galactosidase A enzyme activity in males and confirmed by sequencing the GLA gene in all patients (14). All patients underwent a comprehensive clinical and echocardiographic evaluation. Patients with FD were divided in two groups according to the maximal LV wall thickness: those with LVH (defined as LV wall thickness >12 mm, LVH+) and those without LVH (LV wall thickness ≤12 mm, LVH−), who were regarded as genetic positive but phenotype negative (6). HCs identified from the students, nurses, and doctors of our center and matched for age, sex, and cardiovascular risk factors were included.

This study complied with the ethical principles of the Declaration of Helsinki, and it was approved by our local ethics committee. Informed written consent was obtained from all patients to participate in the study.

### Echocardiography

Comprehensive two-dimensional echocardiography was performed in accordance with current guidelines (15), and as previously described (7), by experienced cardiologists (MCM and GI). STE analysis was performed by experienced cardiologists (FG and RL), blinded to patients’ clinical characteristics, using the commercially available software, 2D Cardiac Performance Analysis© by TomTec-Arena TM (TomTec Imaging Systems, Unterschleissheim, Germany). A speckle tracking analysis was performed in all cardiac chambers in accordance with the latest recommendations (16–18). Briefly, to take the measurements of LV-GLS, images from apical four-, two,- and three-chamber views, zoomed on the LV and acquired with frame rates >50 frames/s, were used. The LV endocardial border was traced from an end-systolic frame and automatically tracked throughout the cardiac cycle by the software. The adequacy of tracking was manually verified and the region of interest properly adjusted. LV-GLS was obtained by averaging all segmental strain values and later by averaging the values calculated in each view. For the RV strain analysis, the average values of the longitudinal peak systolic strain from the three segments of the free wall (RV-FWS) and from all six segments of the free wall and septal wall of the RV (RV-GLS) were calculated (16).

LA strains were measured from the apical four-chamber view, and RA strains were measured from the RV-focused apical four-chamber view (16). The three components of atrial strain were identified from the created curves, as follows: reservoir strain was measured as the peak value during the cardiac cycle; contractile strain was assessed during the peak atrial contraction; conduit strain (strain during passive LV filling) was calculated as the difference between reservoir and contractile strains (16). In patients with atrial fibrillation (AF), measurements were obtained by averaging three consecutive cardiac cycles; in this group, atrial strain analysis was limited to the investigation of atrial reservoir and conduit strains, as recommended by guidelines (16) and performed in other studies (7, 19). Strain values are reported as absolute numbers throughout the text. According to current evidence, RA reservoir <25% was considered impaired (20).

### Statistical analysis

Normally distributed continuous variables were presented as mean ± standard deviation and compared between two groups using an unpaired Student's t-test, whereas non-normally distributed data were expressed as median and interquartile range and compared using the Mann–Whitney *U* test. Categorical data were presented as frequencies and percentages, and a comparison between groups was performed by using *χ*^2^ test or Fisher's exact test, as appropriate. One-way analysis of variance with Bonferroni *post-hoc* tests was used to compare three groups (LVH+, LVH−, and HCs) when continuous variables were normally distributed. Alternatively, the Kruskal–Wallis test was performed when continuous variables were not normally distributed, with Bonferroni *post-hoc* correction.

Univariable and multivariable linear logistic regression analyses were performed to identify the clinical and echocardiographic determinants of RA reservoir strain in the overall population. Variables with a significant correlation in the univariable analysis (*p* < 0.05) were further investigated by using a multivariable model. All tests were two-sided, and *p*-values <0.05 were considered statistically significant. All analyses were performed using SPSS statistical software (SPSS version 23, Inc., Chicago, IL, USA).

## Results

### Clinical characteristics

A total of 64 patients with FD and 64 HCs were enrolled in the study. The main clinical characteristics of the overall FD population are reported in Table 1, while Supplementary Table S1 reports the differences between two groups of FD patients, LVH+ and LVH−. As shown, 50% of patients were male, and the mean age was 50 ± 17 years. The majority of the patients had a classic phenotype (71.8%) and were on specific therapy (57.8%). Roughly, a quarter of them were hypertensive (28.1%), only 8% had chronic kidney disease, and three patients previously underwent kidney transplantation. All but six patients (9.4%) were in sinus rhythm at the time of evaluation.

**Table 1:** Clinical characteristics of the Fabry population.

Variable	Overall Fabry population (*n* = 64)
Age (years)	50 ± 17
Female, *n* (%)	32 (50.0)
Classic phenotype, *n* (%)	46 (71.8)
Sinus rhythm, *n* (%)	58 (90.6)
Hypertension, *n* (%)	18 (28.1)
Diabetes, *n* (%)	1 (1.5)
Dyslipidemia, *n* (%)	9 (14.0)
Ischemic HD, *n* (%)	7 (10.9)
NYHA class ≥2, *n* (%)	20 (31.2)
CKD, *n* (%)	5 (7.8)
RRT, *n* (%)	3 (4.6)
AF, *n* (%)	6 (9.3)
PM, *n* (%)	8 (12.5)
Specific therapy, *n* (%)	37 (57.8)
Previous HF, *n* (%)	2 (3.1)
Previous stroke, *n* (%)	3 (4.6)

HD, heart disease; CKD, chronic kidney disease; RRT, renal replacement therapy; AF, atrial fibrillation; PM, pacemaker; HF, heart failure.

### Standard and speckle tracking echocardiography

Echocardiography showed LVH in 33 patients (LVH+, 51.5%). Table S2 shows a comparison between echocardiographic measurements of the FD population vs. HCs. As expected, several differences emerged among them. Patients with FD had increased LV wall (12 IQR: 9–16 mm vs. 9 IQR: 8–10.9 mm, *p* < 0.001) and RV wall thickness values (4.9 ± 1.9 mm vs. 3.4 ± 0.5 mm, *p* < 0.001); 26 patients (40.6%) had right ventricular hypertrophy (RVH), all of whom were in the LVH group. With regard to the atria, patients with FD had increased left atrial volume index (LAVi) values (*p* < 0.001), while RA dimensions were similar between the two groups defined by RA area (15.3 ± 4 vs. 14.3 ± 2.3 cm^2^, *p* = 0.098) or volume index (23.0 ± 8.1 vs. 20.7 ± 5.2 ml/m^2^, *p* = 0.056).

**Figure 1:**
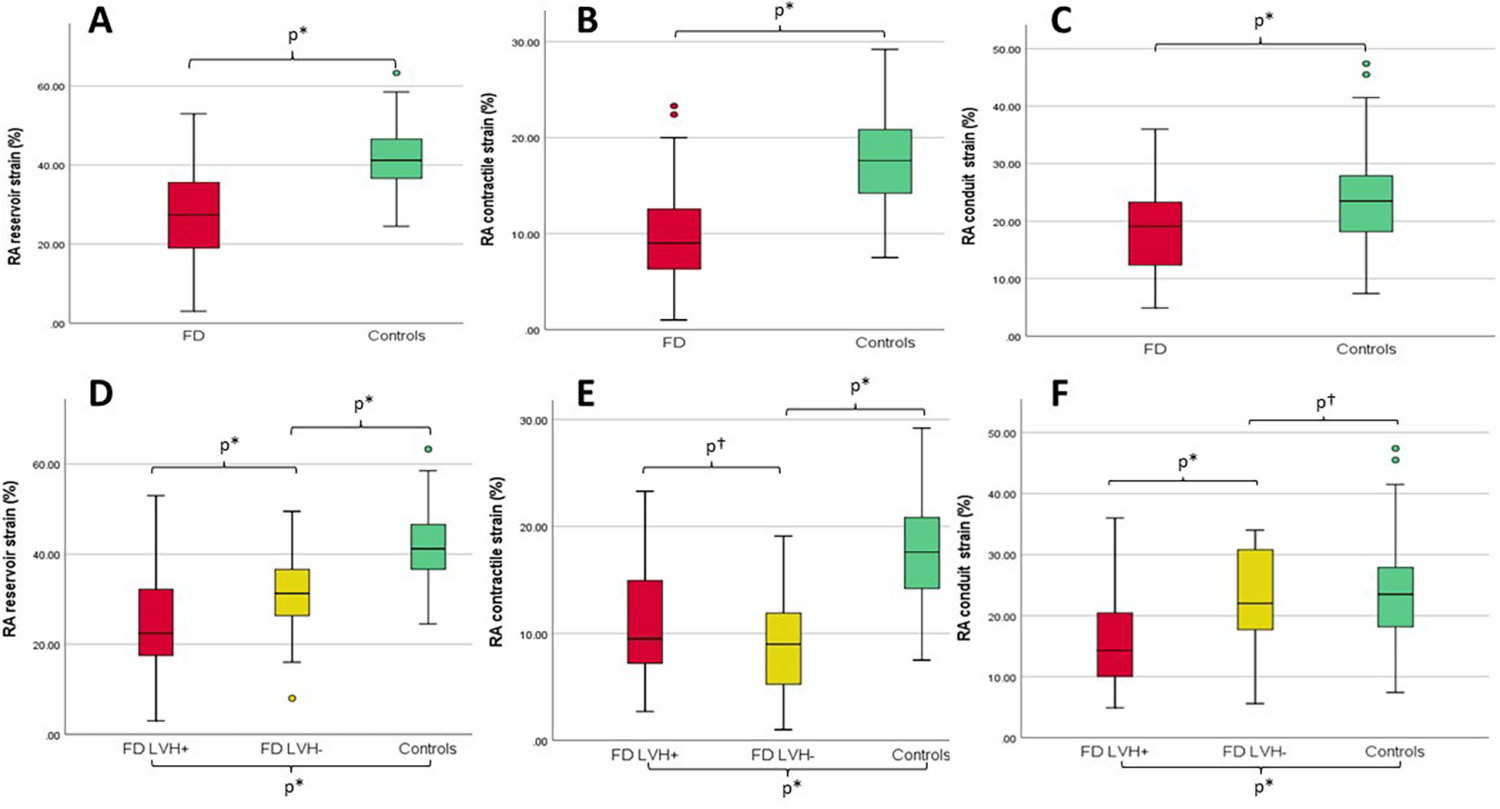
(**A–C**) Box plots with median and interquartile ranges of RA reservoir, contractile, and conduit strains for patients with FD (overall population, red) compared with controls (green), respectively. (**D–F**) Box plots with median and interquartile ranges of right RA reservoir, contractile, and conduit strains for FD patients with LVH (LVH+, red) vs. FD patients without LVH (LVH−, yellow) vs. controls (green), respectively. *p ≤ 0.05; ^†^*p* = not statistically significant; RA, right atrium; LVH, left ventricular hypertrophy.

The Tissue Doppler analysis revealed lower systodiastolic function indices in patients with FD compared with control patients, while LVEF values were similar in both groups. With regard to RV function, RV systolic velocity was lower in patients with FD (12.3 IQR: 11.0–13.7 cm/s vs. 13 IQR: 12–14 cm/s, *p* = 0.041), while tricuspid annular plane systolic excursion (TAPSE) values did not significantly differ between the FD population and HCs (*p* = 0.073), as also right ventricular fractional area change (RVFAC) values (*p* = 0.461). Pulmonary artery systolic pressure (PASP) was higher in patients with FD (even if mostly within normal range, *p* = 0.031), while TAPSE/PASP values were lower (*p* = 0.042). STE analysis revealed impaired values of all chambers strains in patients with FD compared to controls. Specifically with regard to the atria, patients with FD showed lower LA strain (LA reservoir *p* = 0.001, LA conduit *p* = 0.012, LA contractile *p* < 0.001) and RA strain values (RA reservoir: 27.4 ± 11.1% vs. 41.9 ± 8.3%, *p* < 0.001; RA contractile: 9.9 ± 5.1% vs. 18.0 ± 4.9%, *p* < 0.001; RA conduit 19.1 ± 8.1% vs. 24.1 ± 8.1%, *p* = 0.001) when compared with controls (Figure 1); in 27 patients with FD (42.1%), RA reservoir strain was <25%.

Even if FD patients with AF were excluded, all chambers strains were significantly reduced in these patients when compared with controls, as reported in Supplementary Table S3.

The main echocardiographic findings in LVH+ vs. LVH− vs. controls are summarized in Table 2.

**Table 2:** Echocardiographic characteristics of Fabry patients with and without left ventricular hypertrophy and controls.

Variable	LVH+ (*n* = 33)	LVH− (*n* = 31)	Controls (*n* = 64)	*p*-value^a^	*p*-value^b^	*p*-value^c^
Septal WT (mm)	15 (14–19.5)	8.6 (8–10)	9 (8–10.6)	**<0**.**001**	**<0**.**001**	0.999
Posterior LVWT (mm)	13.4 (12.2–15.0)	8 (8–9)	8 (7–9)	**<0**.**001**	**<0**.**001**	0.825
Maximal LVWT (mm)	16 (15–20)	9 (8–10)	9 (8–10.9)	**<0**.**001**	**<0**.**001**	0.999
LVEDD (mm)	48.5 ± 12.3	44.9 ± 5	43.6 ± 4.1	0.253	**0**.**018**	0.999
LVEF (%)	61.6 ± 6.7	63.3 ± 4.3	61.5 ± 3.5	0.473	0.999	0.285
LV-GLS (%)	15.9 (13.8–18.6)	22 (20–23)	23 (22–24)	**<0**.**001**	**<0**.**001**	**0**.**025**
Septal *S*′ (cm/s)	6 (4.6–7)	8 (7.3–8.8)	9 (8–10)	**<** **0**.**001**	**<0**.**001**	0.378
Septal *e*′ (cm/s)	5.6 ± 2.2	11.3 ± 2.6	10.2 ± 2.9	**<0**.**001**	**<0**.**001**	0.189
Septal *a*′ (cm/s)	8 (7–9)	8 (6.5–9)	10 (9–12)	0.999	**0**.**001**	**<0**.**001**
Lateral *S*′ (cm/s)	6.7 (5.6–8.2)	9 (8.1–10.5)	10 (9–11.3)	**<0**.**001**	**<0**.**001**	0.102
Lateral *e*′ (cm/s)	8.3 (7–10.5)	16.2 (13.8–19)	11.1 (10–16.7)	**<0**.**001**	**<0**.**001**	**0**.**010**
Lateral *a*′ (cm/s)	9 (7.3–10)	7 (6.5–9.4)	10 (8–12)	0.477	0.079	**<0**.**001**
E velocity (cm/s)	70 (60.5–80.3)	90 (80–100)	73.5 (66–86)	**<0**.**001**	0.833	**0**.**001**
A velocity (cm/s)	71 (62–86)	55 (50–73)	65 (51.2–78)	0.083	0.152	0.999
E/A	0.9 (0.76–1.22)	1.4 (1.2–1.7)	1.1 (0.8–1.4)	**<0**.**001**	**0**.**013**	**0**.**019**
Average E/e'	10 (7.5–14.5)	7 (6–8)	6.3 (5.5–8)	**<0**.**001**	**<0**.**001**	0.999
LAVi (ml/m^2^)	44.2 (28.3–66.8)	27 (21.3–30.4)	24.9 (21–28.8)	**<0**.**001**	**<0**.**001**	0.250
LA reservoir strain (%)	23.2 (15.9–28.2)	38.8 (28.6–44.0)	35.1 (28.1–44.3)	**<0**.**001**	**<0**.**001**	0.999
LA contractile strain (%)	10.3 (7.2–15.5)	8.5 (6.8–16)	15.4 (11.5–22.4)	0.999	**0**.**004**	**0**.**002**
LA conduit strain (%)	12.7 (10.0–16.8)	16.4 (14–25)	19.2 (13.1–25.3)	**0**.**014**	**0**.**001**	0.999
LL/2 (mm)	29.3 ± 5.2	29.8 ± 4.4	28.3 ± 3.9	0.999	0.925	0.402
TAPSE (mm)	19.9 ± 3.6	23.5 ± 2.9	22.6 ± 2.8	**<0**.**001**	**<0**.**001**	0.768
RV *S*′ (cm/s)	11.7 (9.5–13)	12.5 (12–14)	13 (12–14)	**0**.**024**	**0**.**004**	0.999
RVFAC (%)	42 ± 4.9	43.5 ± 5.3	42. 0 ± 4.1	0.616	0.999	0.539
RV-FWS (%)	19.4 (16.1–25.3)	23 (26–29.2)	23.4 (21.4–29.7)	**0**.**009**	**<0**.**001**	0.484
RV-GLS (%)	17.3 (15.6–22.2)	23.1 (20.1–26.5)	23.4 (21.4–28)	**0**.**001**	**<0**.**001**	0.999
RAA (cm^2^)	17.0 ± 4.7	13.5 ± 2.1	14.3 ± 2.3	**<0**.**001**	**<0**.**001**	0.667
RAVi (ml/m^2^)	25.2 ± 8.3	20.7 ± 7.3	20.7 ± 5.2	**0**.**026**	**0**.**006**	0.999
RA reservoir strain (%)	23.8 ± 11.4	31.3 ± 9.6	41.9 ± 8.3	**0**.**007**	**<0**.**001**	**<0**.**001**
RA contractile strain (%)	11.2 ± 5.7	8.8 ± 4.4	18.0 ± 4.9	0.197	**<0**.**001**	**<0**.**001**
RA conduit strain (%)	15.4 ± 6.9	22.5 ± 7.7	24.1 ± 8.1	**0**.**002**	**<0**.**001**	0.999
PASP (mmHg)	28 (25–30)	25 (20–30)	25 (20–25)	0.763	**0**.**045**	0.894
TAPSE/PAPS (mm/mmHg)	0.72 ± 0.18	0.95 ± 0.23	0.94 ± 0.18	**0**.**009**	**0**.**002**	0.999
RV WT (mm)	6.4 ± 1.8	3.4 ± 0.4	3.4 ± 0.5	**<0**.**001**	**<0**.**001**	0.999

WT, wall thickness; LVWT, left ventricular wall thickness; LVEDD, left ventricular end diastolic dimension; LVEF, left ventricular ejection fraction; LV-GLS, left ventricular global longitudinal strain; LA, left atrium; LAVi, left atrial volume index; LL/2, right ventricle mid diameter; TAPSE, tricuspid annular plane systolic excursion; RV, right ventricle; RVFAC, RV fractional area change; RV-FWS, three-segment right ventricular free wall strain; RV-GLS, six-segment right ventricular global longitudinal strain; RAA, right atrial area; RAVi, right atrial volume index; PASP, pulmonary artery systolic pressure; RV WT, right ventricular wall thickness; *S*′, Tissue Doppler systolic velocity; *e*′, Tissue Doppler early diastolic velocity; *a*′, Tissue Doppler late diastolic velocity.

^b^ Bold values indicate statistically significant *p*-values (<0.05).

^a^ *p-*value for the comparison between Fabry patients with LVH (LVH+) and those without (LVH−).

^b^ *p-*value for the comparison between Fabry patients with LVH (LVH+) and healthy controls.

^c^ *p-*value for the comparison between Fabry patients without LVH (LVH−) and healthy controls.

**Figure 2:**
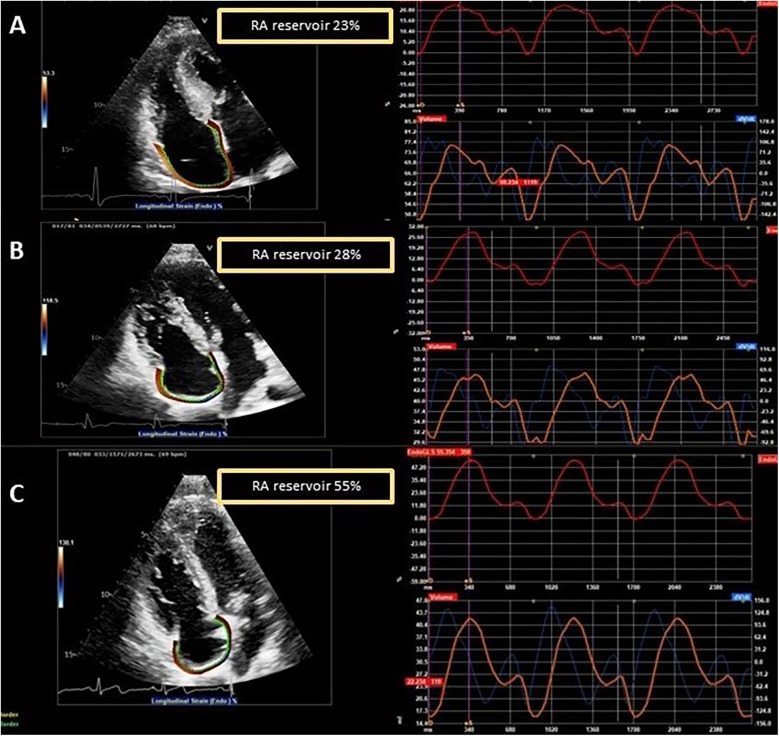
Examples of RA strain assessment in a patient with FD and left ventricular hypertrophy (LVH+, **A**), in a patient with FD without left ventricular hypertrophy (LVH−, **B**), and in a healthy control (**C**).

Among the LVH+ group of patients, the median maximal wall thickness was 16 mm (IQR: 15–20) and, as expected, several differences emerged between the three groups (LVH+ vs. LVH− vs. controls), as shown. Of interest, the LVH− patients differed from controls for lower values of LV-GLS (*p* = 0.025), lateral *e*′ (*p* = 0.010), septal and lateral *a*′ (*p* < 0.001), RA reservoir (31.3 ± 9.6 vs. 41.9 ± 8.3%, *p* < 0.001), and contractile (8.8 ± 4.4 vs. 18.0 ± 4.9%, *p* < 0.001) strains. Figure 2 shows an example of RA strain assessment in a patient with FD LVH+, in a patient with LVH−, and in an HC.

Supplementary Table S4 reports a univariable linear regression analysis for echocardiographic predictors of RA reservoir strain, while a multivariable analysis is presented in Table 3. LA reservoir strain (*p* = 0.010) and LV-GLS (*p* = 0.044) emerged as independent correlates of RA mechanics after adjustments were made for the parameters of RA dimensions, RV systolic function and hypertrophy, and LV maximal wall thickness. No independent predictors of RA reservoir strain have been identified among clinical variables, as reported in Supplementary Table S5.

**Table 3:** Determinants of right atrial reservoir strain in multivariable linear regression analysis.

	Multivariable	
	Beta	*p*-value
RV WT	−1.291	0.193
RV *S*′	0.624	0.145
RAA	−0.494	0.104
Maximal LV WT	0.168	0.680
LA reservoir strain	0.227	**0**.**010**
LV-GLS	0.769	**0**.**044**

RV WT, right ventricular wall thickness; *S*′, Tissue Doppler systolic velocity; RAA: right atrial area; LA, left atrium; LV WT, left ventricular wall thickness; LV-GLS, left ventricular global longitudinal strain.

Bold values indicate statistically significant *p*-values (<0.05).

## Discussion

In this study we investigated RA structural and functional remodeling in patients with FD vs controls. We found that in patients with FD, RA strains were reduced, while RA dimensions were similar between the two groups. Interestingly, RA reservoir and contractile strains were also significantly reduced in patients with FD LVH− when compared with healthy subjects.

Fabry cardiomyopathy is a pan-cardiac disease, as Gb3 accumulates in the lysosomes of all cardiac cellular types (21–23). Indeed, histologic studies have shown that glycosphingolipid deposition also affects atrial cardiomyocytes (24), causing atrial enlargement, impaired function, and a predisposition to supraventricular arrhythmias (25). During the last few decades, studies on patients with FD have mainly focused on *left* atrial involvement, showing that the mean LA size on echocardiography is greater than in age-matched control subjects (2). STE analysis showed that atrial deformation can be impaired even before the occurrence of LVH and diastolic dysfunction, and in patients with overt cardiomyopathy, LA mechanics impairment correlates with the degree of LVH (13, 18).

However, to date, only limited data are available on RA remodeling in FD. Recently, Mattig et al. (19) retrospectively analyzed the diagnostic accuracy of right heart and LA strain parameters to distinguish cardiac amyloidosis (CA) from FD. The authors found that atrial strain parameters were impaired in both patients with CA and those with FD, with patients with CA demonstrating significantly lower LA and RA strain values. Moreover, the authors demonstrated that a combination of standard and STE imaging, including RA strain (together with age, basal RV diameter, and global RV strain), showed the best diagnostic accuracy to distinguish the two diseases.

To the best of our knowledge, this is the first study specifically comparing RA strain in FD patients with and without LVH and controls. The factors that contribute to the impairment of RA mechanics in FD cannot be deduced by the limited data of our study, but we might speculate that the following may have a role: (1) the intrinsic involvement of the RA myocardium, which may affect RA compliance and contractility; (2) the RV systodiastolic dysfunction, affecting RA pressure and right “atrial afterload”; (3) hemodynamic factors influencing RA pressure reliant on LV and LA involvement (26). With regard to the last-mentioned point, the potential role of “left-sided” cardiomyopathy in RA mechanics is supported by the results of the present work, since LA reservoir strain and LV-GLS emerged as independent correlates of RA mechanics after adjustments for the parameters of RA dimensions, RV systolic function and hypertrophy, LV wall thickness.

The findings of this study add new data for the complex understanding of right-sided cardiac involvement in FD. As we demonstrated previously (27, 28), in FD, RV involvement parallels LV structural changes. Indeed, RVH is a feature of advanced disease (14), as suggested by the fact that it is detected in those with concomitant LVH and is associated with the LV mass index and Mainz Severity Score Index (27). Moreover, in our previous work (5), we found that the conventional parameters of RV systolic function, namely, TAPSE, RVFAC, and tissue Doppler imaging systolic velocity, are usually normal even when RVH is present, while 2D-STE is a more sensitive tool to unveil *subtle* RV systolic dysfunction, which turns out to be a common finding even when standard parameters are within normal ranges. Interestingly, in the present work, FD patients without LVH had impaired RA reservoir and contractile strain when compared with controls. Thus, we can speculate that RA strain impairement is an early sign of right-sided FD cardiac involvement. These pieces of evidence support the importance of 2D-STE for a comprehensive echocardiographic evaluation in this disease and the importance of assessing all cardiac chambers strains, including RA.

### Limitations

The main limitation of this work is the small sample size, which carries relevant statistical implications; however, this is a common disadvantage for studies on rare diseases. Moreover, the short follow-up interval and the limited number of major cardiac events did not allow us to assess the clinical and prognostic value of RA strain in FD. Previous studies (29) showed that LA strain is a predictor of AF and LA strain can be more useful in patients with normal LA volumes when compared with those with LA dilatation. In this context, future specifically designed studies aiming to assess the clinical value of RA strain in FD patients with and without RA dilatation could be of great interest.

In the present study, we decided to also include patients with AF and those who had undergone pacemaker implantation previously, with the aim to investigate RA mechanics in a real-world population that included patients with arrhythmias. In the AF group, the investigation was limited to atrial reservoir and conduit strains as recommended by guidelines (16) and performed in other studies on cardiomyopathies (7, 19). As expected, cases of AF and previous pacemaker implantation were found only in FD patients with LVH. Both supraventricular arrhythmias and pacing may alter atrial mechanics, and thus, these factors may have a role in influencing our results.

Lastly, cardiac magnetic resonance data obtained within 1 year from an echocardiographic evaluation were available only in a minority of patients, and hence, the correlation between RA mechanics and tissue characterization could not be assessed.

## Conclusions

In FD, impaired RA strain is a common finding. The results of this study reveal that RA reservoir and contractile strains are reduced in FD patients even before LVH ensues, as compared to controls. LA reservoir strain and LV-GLS show an independent correlation with RA reservoir strain.

## Data Availability

The original contributions presented in the study are included in the article/Supplementary Material, and further inquiries can be directed to the corresponding author.
