# Supplementary material for: Right atrial strain in Anderson–Fabry disease
Source: Front Cardiovasc Med. 2025 Feb 18;12:1496534. doi: 10.3389/fcvm.2025.1496534 (PMC11876412; doi:10.3389/fcvm.2025.1496534)
Supplement: Supplementary file 1 [file Datasheet1.pdf]

Table S1. Clinical characteristics of the Fabry patients with vs without left ventricular hypertrophy.

Variable	LVH + (n=33)	LVH - (n=31)	p-value
Age, years	63 ± 10	36 ± 12	**<0.001**
Female (n, %)	11 (33)	21 (68)	**0.012**
Classic phenotype (n, %)	20 (61)	23 (74)	0.294
AF (n,%)	6 (18)	0	**0.025**
Hypertension (n,%)	15 (45)	3 (10)	**0.002**
Diabetes (n,%)	0	1 (3)	0.484
Dyslipidaemia (n,%)	8 (24)	1 (3)	**0.027**
Ischemic HD (n,%)	7 (21)	0	**0.010**
NYHA class ≥2	16 (48)	4 (13)	**0.003**
CKD (n,%)	5 (15)	0	**0.050**
RRT (n,%)	3 (9)	0	0.239
PM (n,%)	8 (24)	0	**0.005**
Specific therapy (n,%)	24 (73)	13 (42)	**0.022**
Previous HF (n,%)	1 (3)	0	0.999
Previous stroke (n,%)	1 (3)	2 (6)	0.697

AF = atrial fibrillation; CKD = chronic kidney disease; HD = heart disease; LVH = left ventricular hypertrophy; NYHA = New York Heart Association; RRT = renal replacement therapy; PM = pacemaker. Bold values indicate statistically significant p-values (<0.05).

Table S2. Echocardiographic characteristics of the overall Fabry population and controls.

Variable	Overall Fabry population (n=64)	Controls (n=64)	P-value
**Septal WT, mm**	11.2 (8.7-15)	9 (8-10.6)	**<0.001**
**Posterior LVWT, mm**	10 (8-13.4)	8 (7-9)	**<0.001**
**Maximal LVWT, mm**	12 (9-16)	9 (8-10.9)	**<0.001**
**LVEF, %**	62.4 ± 5.7	61.5 ± 3.5	0.300
**LV-GLS, %**	19.1 (15.2-21.8)	23 (22-24)	**<0.001**
**Septal S', cm/s**	7.1 (6-8)	9 (8-10)	**<0.001**
**Septal e', cm/s**	8.3 ± 3.7	10.2 ± 2.9	**0.003**
**Septal a', cm/s**	8 (6.8-9)	10 (9-12)	**<0.001**
**Lateral S', cm/s**	8 (6.7-10)	10 (9-11.3)	**<0.001**
**Lateral e', cm/s**	12.3 (8-16.1)	11.1 (10-16.7)	0.297
**Lateral a', cm/s**	8.1 (7-10)	10 (8-12)	**<0.001**
**E velocity, cm/s**	80 (70-90)	73.5 (66-86)	**<0.001**
**A velocity, cm/s**	67.3 ± 16.1	66.1 ± 16.5	0.672
**E/A**	1.2 (0.8-1.5)	1.1 (0.8-1.4)	0.933
**Average E/e'**	8 (6.5-10.7)	6.3 (5.5-8)	**0.001**
**LAVi, ml/m2**	30.4 (23.6-45.2)	24.9 (21-28.8)	**<0.001**
**LA reservoir strain, %**	28.2 (22.4-40.1)	35.1 (28.1-44.3)	**0.001**
**LA contractile strain, %**	9.8 (7-15.7)	15.4 (11.5-22.4)	**<0.001**
**LA conduit strain, %**	14.9 (11-22.3)	19.2 (13.1-25.3)	**0.012**
**LL/2, mm**	29.5 ± 4.8	28.3 ± 3.9	0.124
**TAPSE, mm**	21.6 ± 3.7	22.6 ± 2.8	0.073
**RV S', cm/s**	12.3 (11.0-13.7)	13 (12-14)	**0.041**
**RVFAC, %**	42.7 ± 5.1	42. 0 ± 4.1	0.461
**RV-FWS, %**	21.4 (18.5-27.0)	24.6 (22.5-29.7)	**<0.001**
**RV-GLS, %**	21.0 (16.4-25.2)	23.4 (21.4-28.0)	**<0.001**
**RAA, cm2**	15.3 ± 4	14.3 ± 2.3	0.098
**RAVi, ml/m2**	23.0 ± 8.1	20.7 ± 5.2	0.056
**RA reservoir strain, %**	27.4 ± 11.1	41.9 ± 8.3	**<0.001**
**RA contractile strain, %**	9.9 ± 5.1	18.0 ± 4.9	**<0.001**
**RA conduit strain , %**	19. 1± 8.1	24.1 ± 8.1	**0.001**
**PASP, mmHg**	25 (23.5-30)	25 (20-25)	**0.031**
**TAPSE/PAPS mm/mmHg**	0.82 ± 0.23	0.94 ± 0.18	**0.042**
**RV WT, mm**	4.9 ± 1.9	3.4 ± 0.5	**<0.001**

WT = wall thickness; LV WT = left ventricular wall thickness; LVEF = left ventricular ejection fraction; LV-GLS = left ventricular global longitudinal strain; LAVi = left atrial volume index; LA = left atrium; LL/2 = right ventricle mid diameter; TAPSE = tricuspid annular plane systolic excursion; RV = right ventricle; RVFAC = RV fractional area change; RV-FWS = 3-segment right ventricular free wall strain; RV-GLS = 6-segment right ventricular global longitudinal strain; RAA = right atrial area; RAVi = right atrial volume; PASP = pulmonary artery systolic pressure; RVWT = right ventricular wall thickness; S'= Tissue Doppler systolic velocity; e'=Tissue Doppler early diastolic velocity; a'=Tissue Doppler late diastolic velocity. Bold values indicate statistically significant p-values (<0.05).

Table S3. Echocardiographic characteristics of the Fabry population excluding patients with atrial fibrillation vs controls.

Variable	Fabry population (n=58)	Controls (n=58)	P-value
**LV-GLS, %**	20.0 (17.1-22.0)	23.0 (22.0-24.0)	**<0.001**
**LA reservoir strain, %**	29.4 (24.5-41.0)	35.4 (28.4-44.8)	**0.007**
**LA contractile strain, %**	9.8 (7-15.7)	15.4 (12.0-22.7)	**<0.001**
**LA conduit strain, %**	15 (11.8-22.6)	19.2 (13.1-25.6)	**0.024**
**RV-FWS, %**	22.2 (19.8-27.9)	24.8 (22.3-29.3)	**0.002**
**RV-GLS, %**	21.8 (17.3-25.6)	23.7 (21.4-28.0)	**0.005**
**RA reservoir strain, %**	29.2 ± 9.9	42.0 ± 8.1	**<0.001**
**RA contractile strain, %**	9.9 ± 5.1	18.1 ± 5.0	**<0.001**
**RA conduit strain , %**	19.4 ± 8.0	24.3 ± 8.4	**0.002**

LV-GLS = left ventricular global longitudinal strain; RV-FWS = 3-segment right ventricular free wall strain; RV-GLS = 6-segment right ventricular global longitudinal strain; RA = right atrial. Bold values indicate statistically significant p-values (<0.05).

Table S4. Echocardiographic determinants of right atrial reservoir strain in univariable linear regression analysis.

	Univariable	
	Beta	P-value
**RV WT**	-0.529	**<0.001**
**RVH**	-0.477	**<0.001**
**TAPSE**	0.324	**<0.001**
**RVFAC**	0.255	**0.004**
**RV S'**	0.326	**<0.001**
**RV-FWS**	0.291	**0.001**
**RV-GLS**	0.286	**0.001**
**RAA**	-0.382	**<0.001**
**RAVi**	-0.353	**<0.001**
**PASP**	-0.240	0.078
**TAPSE/PAPS**	0.264	0.058
**LVEDV**	-0.091	0.307
**LV Maximal WT**	-0.532	**<0.001**
**LVH**	-0.526	**<0.001**
**LAVi**	-0.511	**<0.001**
**E/e'**	-0.426	**<0.001**
**LA reservoir strain**	0.509	**<0.001**
**LA contractile strain**	0.321	**<0.001**
**LA conduit strain**	0.404	**<0.001**
**Septal S'**	0.537	**<0.001**
**Lateral S'**	0.478	**<0.001**
**LVEF, %**	0.150	0.091
**LV-GLS**	0.561	**<0.001**

RVWT = right ventricular wall thickness; RVH: right ventricular hypertrophy; TAPSE = tricuspid annular plane systolic excursion; RVFAC = RV fractional area change; S'= Tissue Doppler systolic velocity; RV-FWS = 3-segment right ventricular free wall strain; RV-GLS = 6-segment right ventricular global longitudinal strain; RAA = right atrial area; RAVi = right atrial volume index; PASP = pulmonary artery systolic pressure; LV WT = left ventricular wall thickness; LVH = left ventricular hypertrophy; LAVi = left atrial volume index; LA = left atrium; LVEF = left ventricular ejection fraction; LV-GLS = left ventricular global longitudinal strain. Bold values indicate statistically significant p-values (<0.05).

Table S5. Clinical determinants of right atrial reservoir strain in univariable and multivariable linear regression analysis.

	Univariable		Multivariable	
	**Beta**	**P-value**	**Beta**	**P-value**
**Age**	-0.350	**0.005**	-0.243	0.069
**Sex**	0.252	**0.045**	0.164	0.178
**NYHA ≥ 2**	-0.277	**0.027**	-0.115	0.385
**Classic phenotype**	-0.107	0.399		
**Hypertension**	0.221	0.080		
**CKD**	-0.319	**0.010**	-0.204	0.100
**Specific Therapy**	0.158	0.212		

CKD = chronic kidney disease; NYHA = New York Heart Association. Bold values indicate statistically significant p-values (<0.05).
